# Effect of *Acacia mearnsii* Tannin Extract Supplementation on Reproductive Performance and Oxidative Status of South African Mutton Merino Rams

**DOI:** 10.3390/ani11113266

**Published:** 2021-11-15

**Authors:** Osman Ahmed, Khoboso Lehloenya, Masindi Mphaphathi, Abubeker Hassen

**Affiliations:** 1 Department of Animal Science, University of Pretoria, Private Bag X20, Hatfield, Pretoria 0028, South Africa; osmanmma@gmail.com; 2 Department of Dairy Production, Faculty of Animal Production, University of Khartoum, P.O. Box 321, Khartoum 11111, Sudan; 3 Department of Agriculture, University of Zululand, Private Bag X1001, KwaDlangezwa 3886, South Africa; khoboso.lehloenya@gmail.com or; 4 Agricultural Research Council, Animal Production, Germplasm Conservation and Reproductive Biotechnologies, Private Bag X2, Pretoria 0062, South Africa; masindim@arc.agric.za

**Keywords:** encapsulated tannin, sheep, testicular measurements, semen quality, testosterone, cortisol, seasonality

## Abstract

**Simple Summary:**

Nutrition and the seasons are two of the better-known variables that affect the reproductive performance of farm animals. In recent years, many antioxidants have been proposed as a tool to improve male reproductive performance. Although such antioxidants, in most cases, are expensive and artificial, tannin extract presents a cheap and natural source of antioxidants. This research evaluated the supplementation effects of tannin extract (TE) and encapsulated tannin extract (ETE) on testicular measurements, semen quality, hormonal status, and oxidative status, as well as the seasonal effect on the testicular measurements of South African Mutton Merino rams. The results suggest that the encapsulation may afford the maximum benefit of TE on sperm concentration and motility. Treatments did not affect the hormonal and oxidative status. The testicular measurements were significantly higher in autumn compared to winter. The plasma level of cortisol correlates negatively with sperm mass motility, progressive motility, viability, and acrosome integrity.

**Abstract:**

We investigated the supplementation effects of *Acacia mearnsii* tannin extract (TE) and encapsulated tannin extract (ETE) on reproductive performance and oxidative status of South African Mutton Merino rams. We also observed the season effect on the testicular measurements. Thirty rams were divided into five groups: 0.0 g TE (control), 1.5 g TE, 3 g TE, 1.5 g ETE, and 3 g ETE supplemented daily for 16 weeks transiting from autumn to winter. Bodyweight and testicular measurements were recorded biweekly. Semen and blood samples were collected weekly during the last five weeks of supplementation. Results showed that the increase in the ETE from 1.5 to 3 g increased the testicular length and sperm concentration, as well as decreased the percentages of low and non-progressive spermatozoa (p<0.05). Simultaneously, the increase in the TE from 1.5 to 3 g decreased semen volume and elevated the percentage of abnormal sperm (p<0.05). The results suggest that the encapsulation of TE affords the maximum benefit of the TE on the sperm quality. Treatments did not affect the hormonal and oxidative status. Testicular measurements were significantly higher in autumn compared to winter. The plasma level of cortisol significantly correlates negatively with sperm motility, viability, and acrosome integrity.

## 1. Introduction

Reactive oxygen species (ROS) are critical for some sperm functions, such as sperm capacitation [1]. However, ROS causes 30% to 80% of male subfertility cases when they exceed the body’s antioxidant capacity [1]. Testicles cells divide at a high rate, consume a high amount of mitochondrial oxygen, and testicles tissues have a high level of unsaturated fatty acids which leave the male reproductive system susceptible to oxidative stress [2]. As a consequence of oxidative stress, ROS may reduce sperm motility and damage the DNA and plasma membrane [3,4]. Antioxidant supplementation is suggested as a tool to break down the oxidative chain reaction and improve the process of spermatogenesis and thereby the sperm quality and general testicles health [1,2]. Numerous antioxidants are proposed to achieve that goal, such as vitamin E, vitamin C, selenium, glutathione, and coenzyme Q10 [5,6]. Although such antioxidants in most cases are expensive and artificial, cheap natural plant antioxidants sources are abundant. A polyflavonoid derived from chains of flavan-3-ol molecules, condensed tannin, represents an inexpensive and promising antioxidant source [7]. For instance, the tannin-rich grape pomace concentrate has shown antioxidant activity equal to vitamin E when it was supplemented into the chicken’s diet [8]. Condensed tannins, extracted from *Ficus altissima* leaves, had protected plasmid DNA and cell against oxidative damage [9]. The addition of commercial tannins extracted from chestnut to the diet of heat-stressed lambs had improved the meat quality and oxidative status of those lambs [10]. The supplementation of tannin-rich *Ficus infectoria* leaf meal had improved the antioxidant status, daily gain and immunity in lambs [11]. However, the potential of tannins to serve as antioxidants must be balanced against their potential to diminish nutrient digestibility and feed intake of the animal [12]. Those negative responses relate to the bitterness and the high tendency of tannin to bind with dietary ruminal protein when a high concentration is consumed [12,13,14]. These limitations can be controlled by reducing the amount of tannin in the diet or/and slowing its release rate in the digestive tract by mean of encapsulation [14]. The encapsulation technique may slow the rate of tannin extract release in the ruminant, prolonging its antioxidant activity. The supplementation of tannin extract should not exceed 0.3 g/kg bodyweight/day (g/kg BW/d) since higher levels may decrease the feed intake and digestibility [15,16]. In South Africa, tannin is mainly extracted from the bark of *Acacia mearnsii*, which is one of the richest sources of tannin [17]. We hypothesised that supplementation of tannin extract would enhance the oxidative status and, thereby, the reproductive performance of the rams. The main objective of this study is to investigate the supplementation effects of *Acacia mearnsii* tannin extract (TE) and encapsulated tannin extract (ETE) on testicular measurements, semen quality, hormonal status, and oxidative status of South African Mutton Merino rams.

Seasonality of reproduction affects the productivity of small ruminants [18]. Photoperiod is the key factor that influences the seasonal effects on the reproduction performance of the rams at high latitudes [19]. However, at lower latitudes, the effect of season on reproduction becomes less and a greater effect for nutrition arises [19]. In addition, besides the individual differences, there are major differences between the breeds in the length of the breeding season and sexual activity [20]. Therefore, the effect of season on testicular measurements and semen quality has been investigated in numerous sheep breeds [21]. As a secondary objective, we aimed to evaluate seasonal variations in the testicular measurements of South African Mutton Merino rams during the transition from autumn to winter. Correlation coefficients between bodyweight, some testicular measurements (testicular volume and scrotal circumference), semen quality parameter (semen volume, sperm concentration, pH, mass motility, progressive motility, viability, acrosome integrity), and hormonal status variables (testosterone and cortisol) are also reported in this study.

## 2. Materials and Methods

### 2.1. Experimental Location and Ethical Clearance

The study was approved by the Animal Ethics Committee (AEC) (Project number: EC056-17) and conducted at the experimental farm of the University of Pretoria in Hatfield, Pretoria, South Africa. The geographical location of the farm was 25${}^{\circ }$44${}^{\prime }$30${}^{\prime \prime }$ S, 28${}^{\circ }$15${}^{\prime }$30${}^{\prime \prime }$ E and 1360 m above mean sea level [22].

### 2.2. Materials

*Acacia mearnsii* tannin extract was obtained from UCL Company (Pty) Ltd. Dalton, South Africa. Tween80, Span80, Dichloromethane (99.9%), N,N-Dimethyl-p- phenylenediamine dihydrochloride (DMPD) and Iron (III) chloride hexahydrate were purchased from (Sigma-Aldrich Ltd., St. Louis, MO, USA).

### 2.3. Encapsulation of the Tannin Extract

The *Acacia mearnsii* tannin extract used in this study composes of 65.8% total phenol, 58.5% total tannin, and 30.5% condensed tannin [23]. The encapsulation of the *Acacia mearnsii* tannin extract was done as previously described by Adejoro et al. [14]. In this process, the particles of tannin extract were micro-encapsulated with palm oil, while Span80 and Tween80 were served as wall materials. Briefly, on a small scale, 8.5 g of the tannin extract was added and then mixed using magnetic starrier to a mixture of 1.5 g palm oil, 0.15 g Span80, and 30 mL Dichloromethane (DCM). The resulting mixture was then gently added to 300 mL of 1.0% (*w*/*v*) aqueous solution of Tween 80 and homogenised for 3 min at 20,000 rpm using a bench-top homogeniser (PRO400DS, Pro Scientific Inc., Oxford, CT, USA). To evaporate the DCM; the resultant emulsion was rotated at 800 rpm for 3 h on a magnetic stirrer. Then the mixture was filtered and washed on a cheesecloth with 100 mL distilled water. The encapsulated tannin microparticles were collected, freeze-dried and stored at 4 ${}^{\circ }$C, until use.

### 2.4. Animals and Treatments

The period of the trial was 16 weeks from autumn to winter (April–August). Thirty Merino rams, aged 12 months old, with an average bodyweight (BW) of 52.78 ± 0.79 kg, were divided randomly into five groups (6 rams/group). The initial bodyweight, testicular parameters, semen volume, pH, colour, as well as sperm concentration, mass motility, and progressive motility were measured for the different groups. All rams were grazed Kikuyu grass (*Pennisetum clandestinum*) during the day (08h00 to 16h00) while kept in sheds during the night. After eight weeks of the trial, the quality of Kikuyu grass dropped drastically; therefore, the rams were offered Eragrostis hay *ad libitum* in the afternoon. The rams were daily supplemented with either 0.0 g tannin extract (control), 1.5 g tannin extract (1.5 g TE), 3 g tannin extract (3 g TE), 1.5 g encapsulated tannin extract (1.5 g ETE), or 3 g encapsulated tannin extract (3 g ETE) for 16 weeks. The daily supplementation of 1.5 g and 3 g of TE or ETE approximately represent 0.03 and 0.06 g/kg BW/day, respectively. The specified amount of supplementation with TE and ETE for each ram was added to 15 g of crushed cornflakes, then mixed with 12 g of 50% water diluted molasses. Then a ball shape was formed from this mixture and provided to the animal fresh every morning. Tannin free balls were provided to the rams in the control group. For ten days before the supplementation period, the rams were trained to consume the balls voluntarily while passing on standardised crash before going to the grazing field.

### 2.5. Bodyweight and Testicular Measurements

The bodyweight and testicular parameters of each ram were taken every two weeks. The bodyweight was measured using an electronic weighing balance (TAL-TEC, South Africa). The testicular measurements were taken after the rams were physically restrained in order to sit their rump on the floor [24]. The scrotal circumference (SC) was measured using a flexible measuring tape placed at the maximum diameter of the scrotal sac, as previously described by Martinez et al. [25]. The width and length of each testicle were measured using a digital vernier graduated in millimetres. When measured, both testicles were force descended into the scrotum, and the epididymis head and tail were not included. The testicular volume (VT) was determined from the means of testicular width (*a*) and length (*b*) using the formula proposed by Steger and Wrobel [26]:

(1) $$VT=\left(1/6\right)\times \pi \times a^{2}\times b\times 0.945\;\left[{\mathrm{cm}}^{3}\right].$$

### 2.6. Ambient Temperature

Data for the ambient temperature were obtained from the Phytotron Department at the experimental farm of the University of Pretoria, Hatfield, Pretoria, South Africa. The average ambient temperatures represented the mean of the temperature—including the maximum and the minimum—throughout the day.

### 2.7. Semen Collection

Semen was collected from rams weekly, starting twelve weeks after the first supplementation of tannin (total of five collections/ram). The 30 rams were divided into six groups of 5 rams in each. Each group (represent all the treatments) was designated to be collected on a certain day of the week. The semen was collected early morning (between 08h00 and 09h00) using an electro-ejaculator (Ramsem, South Africa) with a standardised rectal probe for a small ruminant as previously described by Lukusa and Lehloenya [6]. However, the internal male accessory glands were stimulated through the rectum. The glands were gently massaged using a middle finger of a gloved hand for a few seconds until multiple flehmen responses were obtained. The stimulation was done directly before inserting the rectal probe to perform the electroejaculation process.

### 2.8. Semen Evaluations

#### 2.8.1. Subjective Evaluation

The sperm concentration was estimated using haemocytometer (Hausser, Horsham, PA, USA) [6]. The semen samples were diluted with distilled water 1:400 and the concentration of spermatozoa was calculated as follows:

(2) $$Concentration\;\left(\mathrm{sperm}/\mathrm{mL}\right)=\left(Dilution\;Factor\right)\;\left(Count\;in\;5\;squares\right)\;\left(0.05\times 10^{6}\right)$$

Sperm smears were prepared using nigrosin-eosin stain and the morphological abnormality, viability, and acrosome integrity of the spermatozoa were assessed [27].

#### 2.8.2. Objective Evaluation

Semen samples were transferred in a water bath at 18 ${}^{\circ }$C to the laboratory of Germplasm Conservation and Reproductive Biotechnologies at the Agricultural Research Council (ARC) in Irene. The time taken from the collection site to the laboratory at ARC was 30 min. The sperm total motility (%), speed and progression of the sperm were determined using the Sperm Class Analyzer^®^ system (Microptic, Spain). Semen samples were diluted with Tris-hydroxymethyl aminomethane (1:100) and 5 μL were placed on a pre-warmed glass slide and then mounted with a glass cover-slip before evaluation at a magnification of 10× (Nikon^®^, Shanghai, China).

### 2.9. Blood Collection and Analysis

Five rams from each experimental group were randomly chosen for blood sampling. Blood was sampled from the jugular vein using 18 G BD vacutainer^®^ needle and heparinised BD vacutainer^®^ tubes at day zero, and at the end of the eighth and sixteenth week of the supplementation treatments. The blood samples were centrifuged at 3000 rpm for 10 min and plasma aliquots were harvested and stored at −20 ${}^{\circ }$C until subsequent analysis.

#### 2.9.1. Oxidative Status

Oxidative status was estimated by measuring intermediate products called hydroperoxides which originate from the oxidation of different types of molecules, such as lipids, peptides, and amino acids [28]. The hydroperoxides or the plasma oxidant potential were measured as previously described by Mehdi and Rizvi [29] with minor modifications. Briefly, 100 μL of plasma was added to 1.9 mL N,N-Dimethyl-p-phenylenediamine dihydrochloride (DMPD) (1 mM in acetate buffer). The solution was well mixed, incubated for 10 min and then centrifuged for 5 min at 3000× *g*. Absorbance was recorded at 505 nm against the blank solution and compared with the standard curve of ferric iron for final concentrations. The standard curve of DMPD–ferric iron (0.02–0.20 mM Fe(III)) was generated with highly significant correlation (r=0.998,p<0.001). The concentration of the ferric iron was obtained in accordance with an optical density (absorbance) of 1.00 = 0.137 mM ferric equivalents.

#### 2.9.2. Hormonal Status

Plasma samples were analysed for testosterone and cortisol concentrations using an enzyme immunoassay (EIA) performed on microtiter plates at the endocrine laboratory of the University of Pretoria. A detailed description of the assay component and cross-reactivity has been provided by Palme and Möstl for the cortisol EIA [30] and the testosterone EIA [31]. The sensitivity, intra, and inter-assay variation coefficients of the testosterone EIA used were 0.08 ng/mL, 5.75% to 7.53% and 11.04% to 13.94% respectively. The sensitivity, intra, and inter-assay variation coefficients of the cortisol EIA used were 0.02 ng/mL plasma, 6.21% to 6.98% and 7.01% to 10.35% respectively. Both tests were carried out in duplicates for each sample.

### 2.10. Statistical Analyses

A 2 × 5 factorial model was used to analyse the data on the effects of the season (autumn and winter) and treatments (control, 1.5 g TE, 3 g TE, 1.5 g ETE, and 3 g ETE) on bodyweight and testicular measurements of the rams. Appropriate interactions were estimated, however, these were not significant in most cases; therefore, the main effect means were thus presented. Significant interactions were provided where appropriate. A completely randomised design (CRD) was also used to analyse the effect of treatments on semen quality parameters, hormonal and oxidative status, and the effect of time (day zero, 8 weeks, 16 weeks) on the oxidative and hormonal status. Correlations were estimated using Pearson’s Correlation Coefficient. Means of significant differences were compared using Least Significant Differences (LSD) at a 5% significance level. All the data were conducted using SPSS (IBM SPSS Statistics software 20). Data were expressed as means ± standard error (SE).

## 3. Results and Discussion

Experimental groups were not significantly different in their initial bodyweight, testicular parameters, semen volume, pH, colour, sperm concentration, mass motility, and progressive motility (Table A1). Data on the effect of season and treatments on bodyweight and testicular measurements are presented in Table 1. No significant interactions between treatments and seasons on testicular measurements were found. We observed a significant seasonal effect on the testicular measurements of the rams (Table 1). Mean values of scrotal circumference, testicular volume, testicular width, and testicular length were significantly higher in autumn than those in winter. Authors in previous studies found a similar significant effect for the season on the testicular measurements [32,33,34]. Level of nutrition, daily body growth, and the response of the pineal gland to the day length are the main factors that affect testicular measurements [33]. In this study, the level of nutrition and body growth seems not to be the cause of the observed effect of season on testicular measurements, since the means of bodyweight remind almost the same between the two seasons (Table 1). The longest day in Pretoria was during December and the length of the day starts to decrease during January and February until it gradually becomes the shortest days during June and July [35]. It is, therefore, concluded that the decreasing day length resulted in the secretion of melatonin from the pineal gland [36] which led to increased testicular measurements via the control of the LH and FSH secretion from the pituitary gland [37]. Moreover, we found a significant positive correlation between average ambient temperature and testicular volume (r=0.797,p=0.010) together with scrotal circumference (r=0.813,p=0.008), which support the assumption of the seasonal effect on the testicular measurements. Bodyweight and testicular measurements were not affected by the treatments, except for the testicular length (p= 0.04). The group supplemented with 3 g ETE had a higher testicular length as compared to animals supplemented with 3 g TE and 1.5 ETE (Table 1). Some studies found a positive effect of antioxidant supplementation on the testicular length on rams [38] and goat bucks [39], while others reported no effect on rams [40] and goat bucks [6]. The positive effect of 3 g ETE supplementation on testicular length might be due to its antioxidants properties since the encapsulation process slows and maintains constant release of the tannin microparticles [14]. The variations in reports could be attributed to the differences in the level and duration of the supplementation, and the type of antioxidants used.

Results for the effects of supplementation treatments on semen quality are presented in Table 2. The treatments have a significant effect on semen volume (p=0.042) and sperm concentration (p=0.012). The groups supplemented with 1.5 g TE and 3 g ETE had a higher semen volume as compared to the group supplemented with 3 g TE and a higher sperm concentration as compared to the group supplemented with 1.5 ETE (Table 2). Similar improvements in semen volume and sperm concentration have been reported when antioxidants are supplemented in rams [41,42], goat bucks [43], and rabbits [44]. However, some authors reported no effect of antioxidant supplementation on the semen volume and sperm concentration in rams [45,46] and bulls [47]. Different results following the supplementation with antioxidants on semen volume and sperm concentration could be referred to the variations in the level or the duration of supplementation and the antioxidant type used. Since antioxidants presence increase spermatogenesis [44], the positive effects of 1.5 g TE and 3 g ETE on semen volume and sperm concentration in the present study can be attributed to the potential antioxidant activity in tannin extract. The supplementation with TE and ETE had no significant effect on semen pH and colour (Table 2). In both subjective and objective evaluations, the sperm motility parameters were not influenced by the supplementation treatments, except for the percentages of slow motility (p=0.005) and non-progressive motility (p=0.007). The group supplemented with 3 g ETE had lower percentages of the slow and non-progressive motility spermatozoa as compared to the control, 1.5 ETE and 3 g TE groups. The positive effects of supplementation with antioxidants on sperm motility have been reported in several studies [42,48,49]. The mode of action for the improvement of sperm motility after antioxidant supplementations is still under investigation [50]. However, according to Zhu et al. [51] and the recent review of Barbagallo et al. [50], the antioxidants protect the gene expression system of the spermatozoa from ROS and, thus, maintain ATP generation in the mitochondria, which is important to fuel sperm linear motility [51]. Therefore, the encapsulation of a higher level of tannin extract (3 g ETE) could maintain the antioxidants release, which may protect the ATP generation system in the spermatozoa and thus results in better sperm motility.

**Figure 1:**
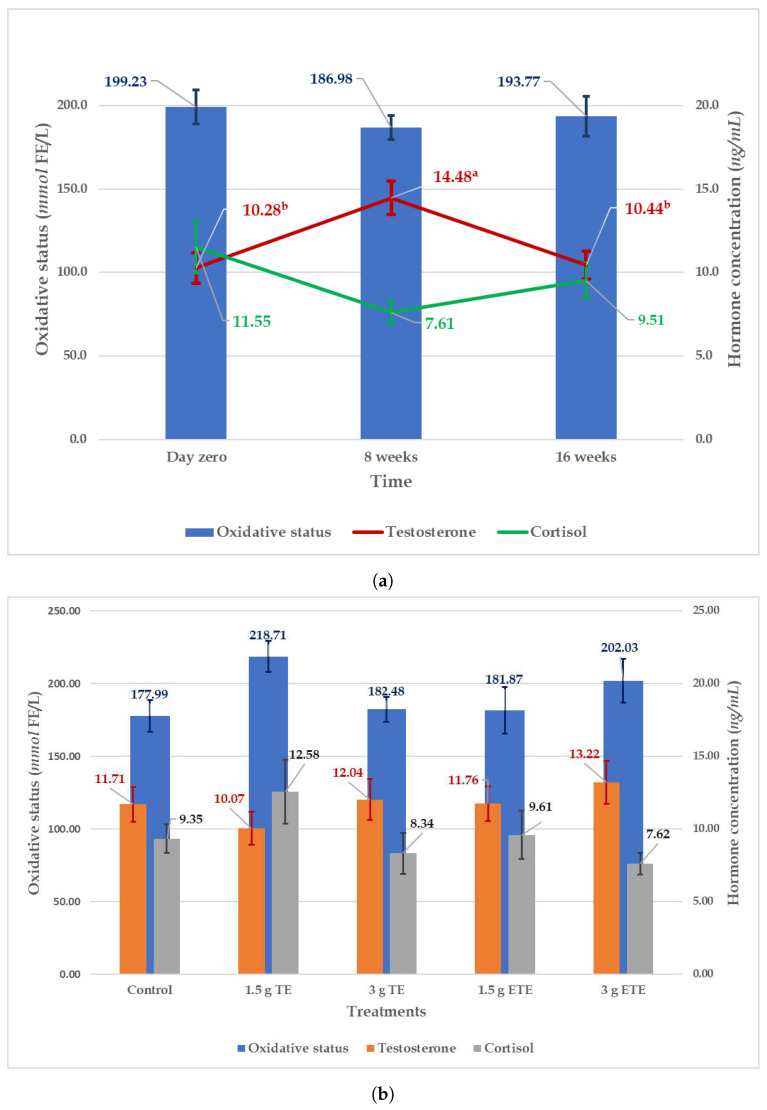
Effect of time (**a**) and supplementation treatments (**b**) on the hormonal and oxidative status (Mean ± SE) of South African Mutton Merino rams. ${}^{a,b}$ Means with different superscripts for the same parameter indicate significant differences (*p* < 0.05). TE = tannin extract; ETE = encapsulated tannin extract.

The effects of supplementation treatments on sperm viability, acrosome integrity, and abnormality are presented in Table 3. The supplementation treatments did not affect sperm viability (p=0.791) and acrosome integrity (p=0.366). However, the supplementation treatments had a significant influence on the sperm total abnormality percentage (Table 3). Rams supplemented with 1.5 g ETE, 3 g ETE, and 1.5 g TE had lower abnormal sperm percentages as compared to those supplemented with 3 g TE. The percentages of the sperm head, midpiece, and tail abnormality were not affected by the supplementation treatment (p=0.777,p=0.124 and p=0.351, respectively), but the sperm cytoplasmic droplet percentage did (p=0.002). The group supplemented with 3 g TE had the highest sperm cytoplasmic droplet percentage. The sperm cytoplasmic droplet is the remnant formed after the phagocytosis of germ cells cytoplasm by the Sertoli cells during spermatogenesis [52]. Incidence of sperm cytoplasmic droplets and disrupted sperm morphology are considered prime features of anomalous spermatozoa leading to oxidative stress in the spermatozoa [53]. In rams, phospholipid-binding protein (PBP) is believed to induce the release of cytoplasmic droplets from epididymal sperm cells during spermatogenesis [54]. The synthesis of PBP was positively associated with the increased level of testosterone in the blood plasma [55]. However, the plasma testosterone level in this study was not affected by the supplementation treatments (Figure 1), it is, therefore, difficult to explain the higher sperm cytoplasmic droplet present in the rams supplemented with 3 g TE.

The effects of supplementation treatments and time (day zero, 8 weeks, and 16 weeks) on the level of male hormone (testosterone), the stress hormone (cortisol) and oxidative status are presented in Figure 1. The supplementation treatments did not affect the oxidative status, testosterone and cortisol. However, despite the research done so far, the mechanism of action that tannins exert on animal tissues is still unknown [56]. The potential antioxidant effect might be a process that took place at the cellular level of the testicles and, therefore, was not detectable in the blood. Future works may consider measuring the oxidative status of the semen or/and testicular tissue. It is worth mentioning here that the 3 g ETE treatment appeared to have the highest level of testosterone and the lowest level of cortisol, but, statistically, was not significant (Figure 1). The plasma testosterone concentration was influenced by time, while the cortisol and the oxidative status were not. At week eight of the study period (Figure 1), the testosterone concentration showed a significant arising trend (14.48 ± 1.00 ng/ml) as compared to day zero (10.28 ± 0.93 ng/ml) and week sixteen (10.44 ± 0.82 ng/ml). Meaning that the testosterone concentration was higher in the second half of autumn (week eight = May) as compared to the first half of autumn (day zero = April) and the end of winter (week sixteen = August). These findings are comparable to the data reported by Sarlos et al. where the plasma concentration of testosterone continued to increase during the first half of autumn until it reached the maximum in the second half and then dropped in winter [57]. The seasonal fluctuations in the plasma testosterone concentration can be attributed to the changes in melatonin secretion due to variation in the daylight length [58,59]. Due to the regulating effect on the hypothalamus–pituitary–testicular axis, melatonin modulates the GnRH pulse activity, and gonadotropin and testosterone production [58].

There were significant interactions between tannin type and tannin supplementation level for testicular length, semen volume, sperm concentration, and percentages of slow motility sperm, non-progressive motility sperm, and sperm with cytoplasmic droplet (Table 4). In most cases, the group of TE at supplementation level of 1.5 g/d and the group of ETE at 3 g/d performed better in comparison with their counterparts (Table 4).

The results in this study revealed an interesting dose-dependent effect of supplementation with TE and ETE on the reproductive performance of the rams. The increase in TE from 1.5 g to 3 g resulted in negative effects in semen volume and sperm morphology. However, on the opposite, the increase in ETE from 1.5 g to 3 g had positive effects on testicular length, sperm concentration and motility. The most likely reason for this disparity is the encapsulation process. As mentioned above, the encapsulation process slows and maintains the constant release of the tannin during the day [14]. Thus, sufficient time may be provided for the environment inside the digestive tract to absorb and utilise the available tannin which could explain the beneficial effects of the supplementation with 3 g ETE. Whereas, in the case of the un-encapsulated tannin extract (TE), the tannin dissolves faster and becomes immediately available [14]. Therefore, the high quantity of the dissolved un-encapsulated tannin (3 g TE) might have worked as an anti-nutritional factor that led to the observed negative effects, since the tannin may combine with the dietary proteins, carbohydrate and minerals and complex with the secreted enzymes and endogenous enzymes, thus diminishing the digestion process [60,61]. However, the low quantity of the dissolved un-encapsulated tannin (1.5 g TE) might be utilised as antioxidants and hence added its beneficial effects. It is crucial to note that the improvements due to supplementation with 1.5 g TE and 3 g ETE did not statistically differ from the control in most cases. This study is the first report on the effect of *Acacia mearnsii* supplementation on the reproduction performance of rams. The daily supplements levels used in our study from TE or ETE were 1.5 g and 3 g per animal, which approximately represent 0.03 and 0.06 g/kg BW/day. These levels are low compared to the dietary tannin inclusion levels used to reduce enteric methane emissions in sheep [62], since we used the amount that is far from the threshold level to identify the least amount of the tannin extract, which can enhance the oxidative status of the rams. As a result, levels higher than 0.06 g/kg BW/day and lower or equal to the threshold level (0.30–0.70 g/kg BW) [15,16,63] were not covered in this study. Those high levels should be considered in future studies in an encapsulated form when provided to animals.

Table 5 shows correlation coefficients between bodyweight, testicular measurements (testicular volume and scrotal circumference), semen quality (semen volume, sperm concentration, pH, mass motility, progressive motility, viability, acrosome integrity), and hormonal status variables (testosterone and cortisol) in Merino rams. Only significant correlations (p<0.05) are discussed. Correlations above r=0.3 are acceptable and considered to have moderate relationship and correlations above r=0.7 are considered to have strong relationship. Bodyweight had a moderate significant correlation with testicular volume (r=0.320,p<0.001), scrotal circumference (r=0.388,p<0.001), and low but significant relationship with semen volume (r=0.266, p=0.012). These results of the positive correlation between bodyweight and testicular measurements agree with the reports from previous studies [64,65,66,67]. The correlation between testicular measurements and bodyweight could be because testicular measurements were relative to bodyweight when age was kept constant [64,67]. Testicular volume had a strong and significant correlation with scrotal circumference (0.728, p<0.001), moderate significant relationship with semen volume (r=0.319, p=0.003) and low but significant correlation with sperm concentration (r=0.257, p=0.016). In addition, the scrotal circumference was found to have a moderate and significant correlation with semen volume (r=0.409,p<0.001) besides low and significant association with sperm concentration (r=0.231, p=0.031) and cortisol concentration (0.295, p=0.010). The positive correlation between testicular measurements (testicular volume plus scrotal circumference) and semen parameters agree with previous studies [65,66,68]. The positive correlation between testicular measurements and semen volume plus sperm concentration could be because seminiferous tubules and germinal cells approximately make-up over 90% of the testicles [69]. The bigger testicles result in more seminiferous tubules and germinal cells which may increase semen production (volume) and sperm concentration [70]. Semen volume showed a low and significant relationship with sperm concentration (r=0.205,p<0.001) and negative, low, and significant correlations with sperm viability (r=−0.243, p=0.004) and sperm acrosome integrity (r=−0.273, p=0.001). A similar positive correlation between semen volume and sperm concentration were reported in previous studies [66,71]. As the spermatogenesis increases, the sperm concentration and semen volume increase and vice versa [72], which may explain the observed positive correlation between semen volume and sperm concentration. Sperm concentration had very low but significant relation with progressive motility (r=0.212, p=0.010). The positive relationship between sperm concentration and progressive motility agrees with the report of Darbandi et al. [73]. Semen pH showed a low, negative but significant association with sperm mass motility and acrosome integrity. A significant negative correlation between sperm motility and semen pH had been reported in different species including sheep [74], buffaloes [75], and humans [76]. The negative correlation between sperm mass motility and semen pH could probably be due to the observation that spermatozoa with high mass motility consume more energy (fructose) and produce more by-products, such as lactic acid [77], which may drop the semen pH faster than those with low mass motility. Sperm mass motility had moderate significant correlations with progressive sperm motility (r=0.421,p<0.001) and sperm acrosome integrity (r=0.327, p<0.001) along with low but significant relationship with sperm viability (r=0.282, p=0.001). The moderate association between sperm mass motility and progressive motility agrees with the finding of Aller et al. [78]. Very low but significant association were found between the progressive sperm motility and viability (r=0.178, p=0.034) plus sperm acrosome integrity (r=0.196, p=0.019). Sperm viability had a moderate positive correlation with sperm acrosome integrity (r=0.608,p<0.001). This could be because spermatozoa gradually lose their acrosome integrity when dies [79]. The cortisol showed a moderate negative correlation with sperm mass motility (r=−0.530, p=0.006), progressive motility (r=−0.437, p=0.029), viability (r=−0.537, p=0.006), and acrosome integrity (r=−0.594, p=0.002). These negative correlations indicate an adverse effect of cortisol on semen quality parameters. This might be because the elevated level of cortisol reduces testosterone secretion [80], which may negatively affect spermatogenesis and semen parameters, such as sperm motility and morphology [81]. In addition, the cortisol leads to an increase in the reactive oxygen species (ROS) [82] that may reduce sperm motility and damage the DNA and plasma membrane [3,4]. However, the negative correlation between cortisol and semen quality parameters seems to differ between the species, as a similar negative correlation was reported in humans [83], while the stallion’s semen seems to be well protected against high levels of cortisol [84].

## 4. Conclusions

To our knowledge, this was the first experimental approach to study the effects of supplementation with encapsulated and non-encapsulated *Acacia mearnsii* tannin extract on oxidative status and reproduction performance of rams. The supplementation with 1.5 g tannin extract (0.03 g/kg BW/d) and 3 g encapsulated tannin extract (0.06 g/kg BW/d) improved the testicular length, semen volume, and sperm concentration, as well as reduced the percentages of spermatozoa with abnormal morphology and low or non-progressive motility. However, the increase in tannin extract supplementation from 0.03 to 0.06 g/kg BW/d reduced semen volume and increased the percentage of abnormal spermatozoa. Thus, to obtain maximum benefits from tannin extract supplementation, the encapsulated tannin extract should be at higher than 0.03 g/kg BW/d. Further studies to elucidate the mechanisms of action that tannin extract has on semen quality and quantity are warranted. Research into the supplementation effect of levels higher than 0.06 g/kg BW/d of encapsulated TE on the oxidative status and reproduction performance of the rams is also needed. Testicular measurements of the South African Mutton Merino rams are higher in autumn than in winter. The plasma level of cortisol may play a significant role in determining the sperm quality of the sheep since it correlates negatively with sperm mass motility, progressive motility, viability, and acrosome integrity.

## Figures and Tables

**Table 1:** Effects of season and tannin supplementation on bodyweight and testicular measurements (Mean ± SE) of South African Mutton Merino rams.

Factors	Parameters				
	Bodyweight (kg)	Scrotal Circumference (cm)	Testicular Volume (cm${}^{3}$)	Testicular Width (mm)	Testicular Length (mm)
**Season**					
Autumn	52.9 ± 0.35	**29.8 ± 0.21 ${}^{a}$**	**150.0 ± 3.44 ${}^{a}$**	**56.5 ± 0.49 ${}^{a}$**	**92.8 ± 0.80 ${}^{a}$**
Winter	52.3 ± 0.41	**28.2 ± 0.20 ${}^{b}$**	**101.2 ± 3.08 ${}^{b}$**	**47.5 ± 0.52 ${}^{b}$**	**87.8 ± 0.88 ${}^{b}$**
***p*** **-value**	0.320	<0.001	<0.001	<0.001	< 0.001
**Treatments**					
Control	52.1 ± 0.69	28.8 ± 0.36	124.2 ± 6.51	51.7 ± 1.07	**90.0 ± 1.42 ${}^{ab}$**
1.5 g TE	52.6 ± 0.77	29.6 ± 0.38	134.3 ± 6.72	53.3 ± 1.03	**91.7 ± 1.53 ${}^{ab}$**
3.0 g TE	51.8 ± 0.38	28.7 ± 0.36	121.6 ± 5.81	51.9 ± 1.00	**88.3 ± 1.23 ${}^{b}$**
1.5 g ETE	52.9 ± 0.42	28.9 ± 0.35	118.5 ± 5.81	51.1 ± 0.97	**88.3 ± 1.31 ${}^{b}$**
3.0 g ETE	53.6 ± 0.60	29.0 ± 0.25	129.4 ± 6.11	52.1 ± 0.94	**93.1 ± 1.23 ${}^{a}$**
***p*** **-value**	0.220	0.299	0.233	0.409	0.040

${}^{a,b}$ Column means with different superscripts within season and treatment indicate significant differences (*p* < 0.05). TE = tannin extract; ETE = encapsulated tannin extract.

**Table 2:** The effect of supplementation treatments on the semen quality parameters (Mean ± SE) of South African Mutton Merino rams.

Variable	Treatments					*p*-Value
	Control	1.5 g TE	3 g TE	1.5 g ETE	3 g ETE	
Semen volume (mL)	**2.0 ± 0.14 ${}^{abc}$**	**2.4 ± 0.20 ${}^{a}$**	**1.6 ± 0.13 ${}^{c}$**	**1.9 ± 0.17 ${}^{bc}$**	**2.2 ± 0.28 ${}^{ab}$**	**0.042**
Semen pH	7.3 ± 0.04	7.3 ± 0.04	7.3 ± 0.07	7.3 ± 0.04	7.3 ± 0.05	0.610
**Subjective evaluation**						
Sperm concentration ($10^{9}$/mL)	**2.9 ± 0.26 ${}^{ab}$**	**3.6 ± 0.31${}^{a}$**	**3.0 ± 0.29 ${}^{ab}$**	**2.3 ± 0.16 ${}^{b}$**	**3.1 ± 0.20 ${}^{a}$**	**0.012**
Semen colour (1–4)	3.8 ± 0.08	3.6 ± 0.15	3.8 ± 0.13	3.7 ± 0.09	3.9 ± 0.06	0.320
Sperm mass motility (1–5)	4.3 ± 0.11	4.3 ± 0.12	4.2 ± 0.13	4.3 ± 0.14	4.2 ± 0.12	0.822
Sperm PM (*%*)	75.0 ± 1.02	75.0 ± 1.75	70.8 ± 1.96	71.1 ± 2.58	73.2 ± 1.90	0.343
**Objective evaluation (SCA^®^)**						
Sperm total motility (*%*)	77.7 ± 1.79	71.7 ± 3.36	74.7 ± 2.82	76.3 ± 2.79	78.6 ± 2.95	0.415
- Rapid motility (*%*)	47.1 ± 2.88	45.1 ± 3.60	44.1 ± 3.73	46.8 ± 3.32	55.9 ± 3.18	0.099
- Medium motility (*%*)	7.1 ± 0.57	6.6 ± 0.54	7.6 ± 0.68	7.5 ± 0.59	5.4 ± 0.65	0.077
- Slow motility (*%*)	**23.6 ± 1.47 ${}^{a}$**	**20.1 ± 1.25 ${}^{ab}$**	**23.0 ± 1.28 ${}^{a}$**	**22.1 ± 1.17 ${}^{a}$**	**17.3 ± 1.31 ${}^{b}$**	**0.005**
Sperm PM (*%*)	50.1 ± 2.80	48.0 ± 3.52	48.0 ± 3.60	50.2 ± 3.30	58.3 ± 3.12	0.143
- Rapid PM (*%*)	29.9 ± 2.36	29.2 ± 2.47	27.8 ± 2.45	28.9 ± 2.02	35.7 ± 2.58	0.154
- Medium PM (*%*)	20.2 ± 1.49	18.8 ± 1.61	20.3 ± 2.13	21.3 ± 1.92	22.7 ± 2.25	0.658
Sperm non-PM (*%*)	**27.7 ± 1.77 ${}^{a}$**	**23.7 ± 1.49 ${}^{ab}$**	**26.7 ± 1.54 ${}^{a}$**	**26.1 ± 1.34 ${}^{a}$**	**20.2 ± 1.67 ${}^{b}$**	**0.007**
Immotiltiy (*%*)	22.3 ± 1.79	28.4 ± 3.36	25.4 ± 2.82	23.7 ± 2.79	21.4 ± 2.95	0.415

${}^{a–c}$ Row means with different superscripts within treatments indicate significant differences (*p* < 0.05). TE = tannin extract; ETE = encapsulated tannin extract; PM = progressive motility; SCA^®^= Sperm Class Analyser.

**Table 3:** The effects of supplementation treatments on sperm viability and morphology (Mean ± SE) of South African Mutton Merino rams.

Variable	Treatments					*p*-Value
	Control	1.5 g TE	3 g TE	1.5 g ETE	3 g ETE	
Viability (%)	72.3 ± 2.77	72.6 ± 2.48	75.5 ± 2.23	72.1 ± 2.81	70.8 ± 3.00	0.791
Acrosome integrity (%)	90.6 ± 0.84	87.9 ± 1.36	86.4 ± 1.49	87.5 ± 2.04	87.0 ± 1.96	0.366
Total abnormalities (%)	**9.2 ± 1.37 ${}^{ab}$**	**7.3 ± 1.54 ${}^{b}$**	**13.1 ± 2.74 ${}^{a}$**	**6.7 ± 0.99 ${}^{b}$**	**6.9 ± 1.36 ${}^{b}$**	**0.048**
Head abnormalities (%)	0.2 ± 0.09	0.3 ± 0.12	0.5 ± 0.31	0.3 ± 0.13	0.2 ± 0.10	0.777
Midpiece abnormalities (%)	5.1 ± 0.70	5.2 ± 1.12	7.3 ± 1.86	4.2 ± 0.63	3.4 ± 0.60	0.124
Tail abnormalities (%)	2.7 ± 0.81	1.4 ± 0.60	2.6 ± 0.76	1.3 ± 0.33	3.0 ± 1.02	0.351
Cytoplasmic droplets (%)	**1.3 ± 0.34 ${}^{b}$**	**0.4 ± 0.18 ${}^{b}$**	**2.7 ± 0.83 ${}^{a}$**	**0.9 ± 0.37 ${}^{b}$**	**0.4 ± 0.17 ${}^{b}$**	**0.002**

${}^{a,b}$ Row means with different superscripts within treatments indicate significant differences (*p* < 0.05). TE = tannin extract; ETE = encapsulated tannin extract.

**Table 4:** Effects of tannin type and tannin supplementation level on the testicular length and some semen quality parameters of Merino rams (Mean ± SE).

Treatments		Parameters					
Tannin Type	Tannin Level (g/day)	Testicular Length (mm)	Semen Volume (mL)	Sperm Concentration ($10^{9}$/mL)	Slow Motility Sperm (%)	Non-Progressive Motility Sperm (%)	Sperm with Cytoplasmic Droplet (%)
TE	1.5	**91.9 ± 1.17 ** ${}^{ab}$	**2.5± 0.61 ** ${}^{a}$	**3.6 ± 0.03 ** ${}^{a}$	**20.1 ± 1.53 ** ${}^{ab}$	**23.7 ± 1.68 ** ${}^{ab}$	**0.4 ± 1.44 ** ${}^{b}$
	3	**88.8 ± 0.95 ** ${}^{b}$	**1.7 ± 0.72 ** ${}^{b}$	**3.1 ± 0.03 ** ${}^{ab}$	**23.0 ± 1.41 ** ${}^{a}$	**26.7 ± 1.58 ** ${}^{a}$	**2.7 ± 2.64 ** ${}^{a}$
ETE	1.5	**88.6 ± 1.02 ** ${}^{b}$	**1.9 ± 0.74 ** ${}^{b}$	**2.4 ± 0.02 ** ${}^{b}$	**22.1 ± 1.32 ** ${}^{a}$	**26.1 ± 1.39 ** ${}^{a}$	**0.9 ± 1.97 ** ${}^{b}$
	3	**93.3 ± 0.93 ** ${}^{a}$	**2.1 ± 0.95 ** ${}^{ab}$	**3.2 ± 0.02 ** ${}^{a}$	**17.3 ± 1.72 ** ${}^{b}$	**20.2 ± 2.03 ** ${}^{b}$	**0.4 ± 1.47 ** ${}^{b}$
**ANOVA**		***p*** **-value**	***p*** **-value**	***p*** **-value**	***p*** **-value**	***p*** **-value**	***p*** **-value**
Tannin Type		0.663	0.830	**0.032**	0.151	0.191	0.051
Tannin level		0.535	0.113	0.585	0.459	0.347	0.068
Tannin type × Tannin level		**0.003**	**0.010**	**0.006**	**0.003**	**0.004**	**0.003**

${}^{a,b}$ Column means with different superscripts within time and treatments indicate significant differences (*p* < 0.05). TE = tannin extract; ETE = encapsulated tannin extract.

**Table 5:** Pearson’s Correlation Coefficient between weight, some testicular measurements, semen quality, and hormonal status variables in the study.

Variable	Bodyweight	Testicular Volume	Scrotal Circumference	Semen Volume	Sperm Concentration	pH	Mass Motility	Progressive Motility	Viability	Acrosome Integrity	Testosterone
**Testicular volume**	0.320 ***	1									
**Scrotal circumference**	0.388 ***	0.728 ***	1								
**Semen volume**	0.266 *	0.319 **	0.409 ***	1							
**Sperm concentration**	0.046	0.257 *	0.231 *	0.205 ***	1						
**pH**	−0.188	−0.175	−0.112	−0.139	−0.056	1					
**Mass motility**	0.030	−0.077	0.001	−0.047	0.017	−0.243 *	1				
**Progressive motility**	−0.052	0.152	0.081	0.104	0.212 *	−0.152	0.421 ***	1			
**Viability**	0.164	−0.061	−0.092	−0.243 **	−0.008	−0.143	0.282 **	0.178 *	1		
**Acrosome integrity**	−0.005	−0.117	−0.078	−0.273 **	−0.006	−0.216 *	0.327 ***	0.196 *	0.608 ***	1	
**Testosterone**	0.205	0.033	0.019	0.202	−0.164	−0.312	0.068	−0.140	0.162	0.165	1
**Cortisol**	−0.028	0.196	0.295 *	0.191	−0.107	0.085	−0.530 **	−0.437 *	−0.537 **	−0.594 **	−0.123

* *p* < 0.05; ** *p* < 0.01; *** *p* < 0.001.

## Data Availability

The data are available from the corresponding author.

## Appendix A

**Table A1:** The initial data of the experimental groups (Mean ± SE) in the study.

Variable	Experimental Groups					*p*-Value
	Control	1.5 g TE	3 g TE	1.5 g ETE	3 g ETE	
Initial Bodyweight (kg)	52.7 ± 1.85	52.3 ± 2.42	52.4 ± 1.59	53.5 ± 1.37	53.0 ± 2.05	0.991
Initial scrotal circumference (cm)	31.6 ± 1.52	31.9 ± 1.39	30.5 ± 0.94	30.7 ± 1.35	30.4 ± 0.52	0.860
Initial testicular volume (cm${}^{3}$)	179.5 ± 22.61	186.3 ± 21.80	182.8 ± 17.93	184.5 ± 15.42	192.8 ± 13.64	0.991
Initial testicular width (mm)	59.7 ± 3.14	61.7 ± 2.47	61.4 ± 2.25	62.4 ± 2.40	61.7 ± 1.49	0.951
Initial testicular length (mm)	98.8 ± 4.40	96.9 ± 4.57	96.8 ± 4.17	97.5 ± 2.52	99.0 ± 4.26	0.992
Initial semen volume (mL)	0.7 ± 0.11	0.7 ± 0.18	1.0 ± 0.14	0.9 ± 0.23	0.8 ± 0.19	0.728
Intial semen pH	5.8 ± 0.17	5.9 ± 0.37	5.7 ± 0.33	6.5 ± 0.62	6.5 ± 0.22	0.355
Initial sperm concentration (10${}^{9}$/mL)	3.8 ± 0.90	3.9 ± 0.75	3.9 ± 0.69	3.9 ± 0.91	3.5 ± 0.80	0.997
Initial Semen colour (1–4)	3.2 ± 0.31	3.2 ± 0.48	3.7 ± 0.21	2.8 ± 0.40	3.5 ± 0.22	0.471
Initial Sperm mass motility (1–5)	3.3 ± 0.21	3.3 ± 0.56	3.5 ± 0.43	3.3 ± 0.61	3.8 ± 0.17	0.907
Initial progressive motility (%)	56.2 ± 4.86	54.5 ± 10.80	52.2 ± 9.31	51.1 ± 12.71	56.4 ± 6.71	0.991

TE = tannin extract; ETE = encapsulated tannin extract.
